# Assessment of Risk Factors for Fractures in Patients with Type 2 Diabetes over 60 Years Old: A Cross-Sectional Study from Northeast China

**DOI:** 10.1155/2020/1508258

**Published:** 2020-01-27

**Authors:** Yan Guo, Yingfang Wang, Feng Chen, Jiabei Wang, Difei Wang

**Affiliations:** Department of Geriatrics, The First Affiliated Hospital of China Medical University, Shenyang, Liaoning 110001, China

## Abstract

**Aims:**

Previous evidence has demonstrated an increased fracture risk among the population with type 2 diabetes mellitus (T2DM). This study investigated the prevalence of bone fractures in elderly subjects (with and without type 2 diabetes) and identified any fracture risk factors, especially the risk factors for common known fractures in particular diabetic populations.

**Methods:**

This cross-sectional study was conducted with community-dwelling people over 60 years old in nine communities from the city of Shenyang, which is the capital of Northeast China's Liaoning Province. A total of 3430 elderly adults (2201 females, mean ± standard deviation age 68.16 ± 6.1 years; 1229 males, 69.16 ± 6.7 years) were included. Our study measured the heel bone mineral density (BMD) and used the timed “up and go” (TUG) test and other indicators. In addition, we performed logistic regression analysis to explore the risk factors for fractures in the general population and the diabetic population and to analyze the differences.

**Results:**

The results revealed that a total of 201 elderly persons (5.8%), with an average age of 70.05 ± 6.54 years, suffered from a history of fragility fractures, which affected more females (74.6%) than males (*p* = 0.001). The prevalence of fractures in the T2DM population was 7.3%, which was much higher than the 5.2% in non-T2DM population (*p* = 0.001). The prevalence of fractures in the T2DM population was 7.3%, which was much higher than the 5.2% in non-T2DM population (*p* = 0.001). The prevalence of fractures in the T2DM population was 7.3%, which was much higher than the 5.2% in non-T2DM population (*p* = 0.001). The prevalence of fractures in the T2DM population was 7.3%, which was much higher than the 5.2% in non-T2DM population (*T*‐score≤−2.5 (OR 1.750) were independent risk factors for fragility fractures in the non-T2DM population, but they were not risk factors in the T2DM population.

**Conclusions:**

This study found that low BMD and slow TUG time were independent risk factors for fractures in non-T2DM patients, while no associations were found in the T2DM population. Patients with T2DM have a higher risk for fractures even when they have sufficient BMD and a short TUG time. TUG and BMD underestimated the risk for fractures in the T2DM population.

## 1. Introduction

The prevalence rate of type 2 diabetes mellitus (T2DM) has increased to 18% (aged 65-99) around the world in 2017 [1], and the prevalence of T2DM in China's elderly population has increased along with the aging of the Chinese population and the rise in unhealthy lifestyle habits, and environmental pollution [2]. Individuals with T2DM have a higher risk for fractures than those without T2DM, but epidemiological data are limited [3–5]. Fractures seriously affect the quality of life, and many more prediction methods that are simple and practicable should be explored. Bone strength includes not only bone density but also bone quality, and it is typically used as a measure of skeletal disorders that are associated with fractures [6]. Most studies have revealed that bone mineral density (BMD) is not lower in patients with T2DM, and in fact, it is higher than that in non-T2DM persons [6–8], but the cause of this phenomenon is not yet clear. The timed “up and go” (TUG) test was originally described by Podsiadlo as a mobility test for frail older persons [9]. Some studies have shown that the TUG test is a sensitive and specific measure for community-dwelling adults in predicting falls and an independent risk factor for fracture [10, 11], but the relationship between TUG and fracture risk in T2DM-specific populations is unclear. Thus, it is of great interest to us to evaluate whether the TUG results could predict fractures in diabetic populations. We performed a cross-sectional study to investigate the prevalence of fractures in a population of 3430 elderly subjects (with and without type 2 diabetes) in order to identify fracture risk factors and, in particular, to assess the risk factors for common fractures in particular diabetic populations.

## 2. Materials and Methods

### 2.1. Study Population

We conducted a cross-sectional study of permanent residents over 60 in nine communities in Shenyang, Northeast China, from May to October 2017. The exclusion criteria were as follows: secondary osteoporosis; cancer; glomerular nephritis, or creatinine clearance (Ccr) < 30 mL/min; hyperthyroidism or hypothyroidism; and previous diagnosis of osteoporosis and treatment. A total of 3430 seniors took part in our survey after removing incomplete samples, including 1073 suffering from T2DM and 201 samples suffering from fragility fractures. We used a stricter standard for fragility fractures in the population who had fractures caused by a minor crash or fall and obtained a definite diagnosis from a clinician without distinguishing the fracture location.

This study was approved by the ethics committee of the First Affiliated Hospital of China Medical University and was conducted in accordance with the principles described in the Declaration of Helsinki. All subjects provided written informed consent prior to participation. The research has been registered on the Chinese Clinical Trials Registry (ChiCTR-ERC-17011100).

### 2.2. Clinical Data Collection

All subjects were assessed with a standardized questionnaire based on the Community Health Questionnaire administered by trained doctors, including basic demographics, history of present illness, past medical history, lifestyle risk factors such as smoking and alcohol consumption, and medication used. Each subject was examined for height, weight, waist circumference (WC), and hip circumference (HC); each measurement was evaluated twice and then averaged (accurate to 0.1 cm). Body mass index (BMI) was calculated with the following equation: weight (kg)/(height (m^2^)). The waist to hip ratio (WHR) was the ratio of WC (cm) to HC (cm). Systolic pressure (SBP) and diastolic pressure (DBP) were also measured twice and averaged.

### 2.3. Biochemical Measurements

Blood samples were collected following overnight fasting. Serum fasting blood glucose (FBG), glycosylated hemoglobin (HbA1c), uric acid (UA), total cholesterol (TC), triglyceride (TG), high-density lipoprotein cholesterol (HDL-C), low-density lipoprotein cholesterol (LDL-C), creatinine (Cr), serum, and calcium (Ca) were measured by an automatic biochemical analyzer, and 25-hydroxyvitamin D_3_ (25 (OH)D_3_) was measured by mass spectrometry. And Ccr was calculated using the formula from Cockcroft and Gault [12].

### 2.4. TUG

The timed “up and go” (TUG) test records the time it takes to rise from an armed chair, walk 3 meters, and return to sit in the chair. In this study, a TUG result > 10.2 s was defined as poor mobility, according to a previous study [9].

### 2.5. BMD

The heel BMD was measured by an ultrasonic bone densitometer (Hologic Sahara ultrasound bone density densitometer, software: version3.1, American Hologic Corporation), which has good correlations with dual-energy X-ray absorption (DXA) measurement. In this study, we defined normal density, osteopenia, and osteoporosis (OP) as a *T*‐score≥−1.0, between -1.0 and -2.5, and ≤-2.5, respectively, following the World Health Organization definitions [13].

### 2.6. Statistical Analysis

All statistical analyses were performed with Statistical Package for the Social Sciences version 20.0 (SPSS Inc., Chicago, IL, USA), and significant differences were assumed to be present at *p* < 0.05 (two tailed). Data are expressed as the mean ± SD for continuous variables or percentages (%) for categorical variables. *t*-tests for continuous variables or chi-square test for categorical variables was used to compare parameters between two groups. Logistic regression analysis was performed to identify risk factors.

## 3. Results

### 3.1. Baseline Characteristics and Laboratory Parameters

The baseline characteristics and laboratory parameters of this study population were stratified by a history of fragility fractures into fracture and nonfracture groups (Table 1). There were 3430 people in this study, of which 2201 (64.2%) were female and 1229 (35.8%) were male, with ages ranging from 60 to 92 years old. A total of 201 subjects (5.8%) suffered from a history of fragility fractures. Compared with the nonfracture group, the fracture group was older (70.05 ± 6.54 vs. 68.43 ± 6.30, *p* < 0.001) and included more females (74.6% vs. 63.5%, *p* = 0.001). TUG and BMD were measured in the study population. The BMD was found to be much lower and the TUG time much longer in the fracture group than in the nonfracture group (*p* < 0.001). In the fracture group, the prevalence rate of T2DM was 38.8%, which was much higher than that in the nonfracture group (*p* < 0.05).

### 3.2. The Risk for Fracture

We performed a logistic regression analysis for the total population and found that diabetes was a risk factor for fracture (OR 1.357). Moreover, the female sex (OR 1.663), older age (OR 1.026), slow TUG time (OR 1.454), and osteoporosis (OR 1.799) were risk factors for fractures after adjusting for the confounding factors in this study (Table 2).

### 3.3. The Baseline Characteristics and Risk Factors Stratified by T2DM and Fracture

We divided all subjects into a diabetes population and a nondiabetic population and analyzed the differences in sex, age, uric acid, Ccr, FBG, HbA1c, 25(OH)D_3_, Ca, WHR, BMI, BMD, and TUG between two populations (Table 3). In our study, the prevalence rate of T2DM was 31.3%, and the prevalence of hypertension rate was 56.5% (not shown). The BMD was similar between the two populations, while the TUG time was longer in the T2DM population than in the non-T2DM population.

**Figure 1:**
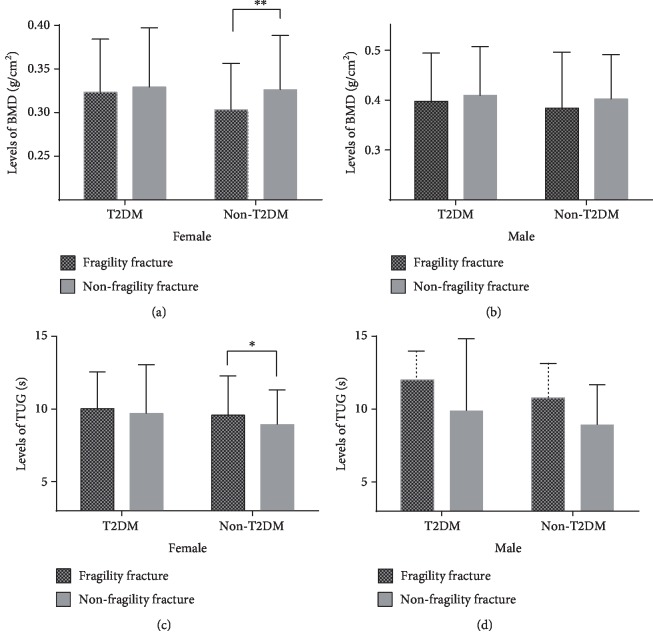
(a) Levels of BMD in different groups for female. (b) Levels of BMD in different groups for male. (c) Levels of TUG in different groups for female. (d) Levels of TUG in different groups for male. ^∗∗^*p* < 0.001, ^∗^*p* < 0.05.

We divided all subjects into a diabetic population and a nondiabetic population and analyzed the differences in sex, age, UA, Ccr, FBG, HbA1c, 25 (OH)D_3_, Ca, WHR, BMD, and TUG time between the fracture group and nonfracture group for the two populations. We found that sex, age, UA, BMD, and TUG time were different between the fracture group and the nonfracture group in the nondiabetic population. However, we did not find significant differences in TUG time and BMD between the fracture group and the nonfracture group in the diabetic population (Table 4). The differences in TUG and BMD values between the fracture and nonfracture groups in diabetic and nondiabetic populations were stratified by sex, and the differences in TUG and BMD values in nondiabetic women were found to be statistically significant (Figures 1(a)–1(d)). We used logistic regression analysis to assess the relationship between fractures and multiple risk factors in the T2DM and non-T2DM populations, and the results are shown in Table 5. The female sex (OR 1.835), TUG time > 10.2 s (OR 1.602), and *T*‐score≤−2.5 (OR 1.750) were found to be independent risk factors for fragility fractures in the nondiabetic population but not in the T2DM population after adjusting for confounding factors (Table 5).

### 3.4. Multiple Metabolic Parameters May Affect TUG Time and BMD in the Diabetic Population

We stratified the diabetic population into groups according to TUG time > 10.2 s and *T*‐score≤−2.5. We analyzed the relationships among TUG time, BMD, and multiple metabolic parameters that are usually combined with diabetes, including age, BMI, SBP, DBP, HbA1c, Ccr, UA, TC, TG, HDL-C, LDL-C, Ca, 25 (OH)D_3_, history of HTN, and T2DM therapy. TUG time and *T*-score were found to be related to multiple factors (Table 6) that may influence the results showing that TUG time and BMD are not independent risk factors for fragility fractures in the diabetic population.

## 4. Discussion

This study population was from a high latitude region in China with cold weather, low levels of sunlight, and high risk for slips and falls in the winter. In addition, the age of the population was over 60 years old, which is considered retirement age and is defined as geriatric in China. The sample size and demographic characteristics of the population in this study differ from those in other studies.

Previous studies have researched multiple risk factors for fragility fractures, such as vitamin D intake or serum concentration [14], level of UA [15], obesity or low weight [16, 17], body composition [18], osteoporosis, and falls. Individuals with T2DM and type 1 diabetes mellitus (T1DM) have a higher risk for fractures, particularly hip fractures, than nondiabetic subjects, including both men and women [5, 19]. Previous studies have shown that the risks of fractures in those diagnosed with diabetes were higher than those in non-Hispanic black (HR 1.86 (95% CI 1.05–3.30)) and Mexican American (HR 2.29 (95% CI 1.41–3.73)) adults without diabetes [20]. T2DM factors such as a longer disease duration [21], diabetic complications, poor glycemic control [22], insulin resistance (IR) [23], and the use of insulin or oral antidiabetic medication [19, 24] have a complex pathophysiological interaction with fractures. And an increased risk for falls were also reported to increase the fracture risk [25].

Our study found that the prevalence rate of fragility fractures in the diabetic population was 7.3%, which is much higher than the 5.2% in the non-T2DM population (*p* = 0.018, Table 1), and analyzed the association between fractures and diabetes, hypertension (HTN), HbA1c, blood lipids, BMI, level of serum 25 (OH)D_3_, insulin use, oral antidiabetic medication, Ccr, etc. We found that T2DM, female sex, older age, slower TUG time, and osteoporosis are risk factors for fractures.

BMD is measured to assess osteoporosis in many medical institutions. Although previous studies have reported that the BMD of patients with T2DM was normal or even higher than that of nondiabetic controls, the fracture risk was higher in patients with T2DM [4, 26]. Our study also showed that the BMD levels between T2DM and non-T2DM patients were similar (*p* = 0.131), although the T2DM population had a higher risk for fractures (OR 1.357).

The TUG test is usually performed to reflect the risk for falls and fractures [6, 11, 27]. D.C.C. de Abreu et al.'s [28] research reported that the TUG times did not present correlations with fall's history, which reminds us that a slow TUG time may be related to a higher risk for fractures but did not totally account for the higher fall risk; some other aspects may also be involved. The TUG test could reflect muscle strength, impaired gait and balance, and increased fall risk, which are associated with the risk for fractures, especially in geriatric patients [27].

Mousa et al. [27] conducted a case-control study of 138 elderly individuals aged over 60 years from a hospital who showed abnormal TUG times; a TUG time > 20 s was defined as poor mobility and was strongly associated with a reduced BMD and increased fracture risk, but the study did not analyze the T2DM population in particular.

In this study, we defined TUG times > 10.2 s as poor mobility according to Podsiadlo and Richardson [9]. The populations were from communities and were in better physical condition than populations from medical institutions, and the mean TUG times were lower than 10 s. Thus, the cutoff point of <10.2 s was highly suitable for study inclusion.

The risk for fractures in the diabetic population is much higher than that in the nondiabetic population, so we wondered whether other risk factors of fractures differ between T2DM and non-T2DM populations. We divided all subjects into T2DM and non-T2DM populations and analyzed the differences in risk factors for fracture, including but not limited to TUG times and BMD. We found that, after adjusting for confounding factors, a slow TUG time and lower BMD were risk factors for fracture in the nondiabetic population but not the diabetic population, which is quite interesting. What influenced the difference between the two population types? Diabetic patients often have multiple metabolic diseases and take multiple medications. Were there some confounding factors of diabetes that influence the specificity? We therefore explored the factors that may affect TUG time and BMD in the T2DM population that are usually associated with diabetes. As shown in Table 5, we found several confounding factors that are involved, such as hypertension, Ccr, BMI, and 25 (OH)D_3_. Since the data we collected could not represent all the abnormalities in people with diabetes, we did not perform further analyses on what factors exactly affect TUG time and BMD.

At present, many specialists have recognized that BMD does not predict the risk for fractures in diabetic patients very well, which is similar to our results, but there is a lack of understanding of the predictive value of the TUG time. Clinically, many doctors use the TUG test to predict the risk for fractures in diabetes. However, our study showed that individuals with T2DM and fractures may have a good TUG test time but a high risk for fractures. We still accept that the TUG test can reflect a person's physical state, but it is not appropriate for screening the risk for fractures in T2DM patients.

There were some limitations in this study. First, the study design was cross-sectional. But this was not a cohort study and could not follow the outcomes of the patients. Therefore, more prospective studies with intervention strategies are needed to verify our results. Second, our study did not distinguish the fracture location because the data were incomplete. Third, we roughly collected the therapy methods for diabetes, but not in detail. The ages of the participants were all above 60 years, but not all ages may have the same conclusion.

## 5. Conclusion

This study found that low BMD and slow TUG time were independent risk factors for fractures in non-T2DM patients, while no associations were found in the T2DM population. Patients with T2DM have a higher risk for fractures even when they have sufficient BMD and a short TUG time. TUG and BMD underestimated the risk for fractures in the T2DM population.

## Figures and Tables

**Table 1:** Baseline characteristics and laboratory parameters of total population stratified by fracture.

Variables	Fracture (*n* = 201, 5.8%)	Nonfracture (*n* = 3229, 94.2%)	*p* value
Sex, female (%)	150 (74.6)	2051 (63.5)	0.001^∗^
Age (years)	70.05 ± 6.54	68.43 ± 6.30	<0.001^∗∗^
Height (cm)	159.87 ± 8.06	160.99 ± 8.15	0.058
Weight (kg)	63.13 ± 9.76	64.50 ± 10.44	0.07
BMI (kg/m^2^)	24.71 ± 3.37	24.84 ± 3.27	0.572
WC (cm)	88.66 ± 8.96	87.33 ± 9.36	0.17
HC (cm)	97.94 ± 7.16	97.94 ± 7.16	0.873
SBP (mmHg)	140.01 ± 19.62	138.63 ± 20.42	0.826
DBP (mmHg)	81.37 ± 11.59	80.13 ± 11.79	0.878
WHR	0.9 ± 0.06	0.89 ± 0.06	0.015^∗^
BMD (g/cm^2^)	0.33 ± 0.08	0.36 ± 0.08	<0.001^∗∗^
TUG (seconds)	10.14 ± 4.55	9.18 ± 3.09	<0.001^∗∗^
HbA1c (%)	6.11 ± 1.47	5.9 ± 1.17	0.075
FBG (mmol/L)	6.44 ± 2.23	6.09 ± 1.76	0.023^∗^
UA (*μ*mol/L)	292.23 ± 93.75	303.58 ± 102.49	0.126
Ccr (mL/min)	73.10 ± 19.65	75.56 ± 21.40	0.114
TC (mmol/L)	5.06 ± 0.95	5.12 ± 1.02	0.388
TG (mmol/L)	1.69 ± 1.03	1.80 ± 5.33	0.776
HDL-C (mmol/L)	1.39 ± 0.38	1.37 ± 0.42	0.4
LDL-C (mmol/L)	3.07 ± 0.79	3.16 ± 0.91	0.165
Ca (mmol/L)	2.41 ± 0.1	2.42 ± 0.09	0.258
25 (OH)D_3_ (ng/mL)	22.38 ± 7.93	22.19 ± 7.62	0.735
History of hypertension (%)	101 (50.2)	1391 (43.1)	0.047^∗^
History of T2DM (%)	78 (38.8)	995 (30.8)	0.018^∗^
History of smoking (%)			0.126
Never	173 (86)	2591 (80.2)	
Current	18 (9)	422 (13.1)	
Quit	10 (5)	216 (6.7)	
Current drinking (%)			0.24
Never	165 (82.1)	2498 (77.4)	
Current	30 (14.9)	639 (19.8)	
Quit	6 (3)	92 (2.8)	
Therapy of T2DM			0.49
Oral	10 (12.8)	198 (19.9)	
Insulin	7 (9)	87 (8.7)	
Oral and insulin	3 (3.8)	30 (3.1)	
Without medicine	58 (74.4)	680 (68.3)	

Abbreviations: BMI: body mass index; WC: waist circumference; HC: hip circumference; WHR: waist hip ratio; SBP: systolic pressure; DBP: diastolic pressure; HbA1c: glycosylated hemoglobin; FBG: fasting blood glucose; UA: serum uric acid; Ccr: creatinine clearance; TC: total cholesterol; TG: triglycerides; HDL-C: high-density lipoprotein cholesterol; LDL-C: low-density lipoprotein cholesterol; 25 (OH)D_3_: 25-hydroxyvitamin D3; BMD: bone mineral density; TUG: timed “up and go.” ^∗∗^*p* < 0.001, ^∗^*p* < 0.05.

**Table 2:** Analysis of risk factors for fracture in all subjects.

Variables	OR	95% CI	*p* value
Sex, female	1.663	1.192-2.312	0.003^∗^
Age (years)	1.026	1.002-1.050	0.032^∗^
TUG > 10.2 s	1.454	1.040-2.032	0.029^∗^
History of T2DM	1.357	1.008-1.826	0.044^∗^
*T*-scores			
Normal	1		
Osteopenia	1.261	0.744-2.137	0.389
Osteoporosis	1.799	1.072-3.02	0.026^∗^

Abbreviations: TUG: timed “up and go.” ^∗^*p* < 0.05.

**Table 3:** Baseline characteristics and laboratory parameters stratified by T2DM.

Variables	T2DM (*n* = 1073, 31.3%)	Non-T2DM (*n* = 2357, 68.7%)	*p* value
Sex, female (%)	691 (64.4)	1510 (64.1)	0.85
Age (years)	69.09 ± 6.53	68.26 ± 6.21	<0.001^∗∗^
UA (*μ*mol/L)	321.37 ± 86.6	311.49 ± 83.13	0.002^∗^
Ccr (mL/min)	76.46 ± 21.51	74.94 ± 21.2	0.054
FBG (mmol/L)	7.65 ± 2.43	5.4 ± 0.6	<0.001^∗∗^
HbA1c (%)	6.96 ± 1.47	5.44 ± 0.58	<0.001^∗∗^
25 (OH)D_3_ (ng/mL)	22.19 ± 7.62	22.21 ± 7.65	0.95
Ca (mmol/L)	2.42 ± 0.09	2.41 ± 0.09	0.001^∗^
WHR	0.91 ± 0.06	0.89 ± 0.06	<0.001^∗∗^
BMI (kg/m^2^)	25.29 ± 3.31	24.63 ± 3.25	<0.001^∗∗^
BMD (g/cm^2^)	0.36 ± 0.09	0.35 ± 0.08	0.131
TUG (s)	9.82 ± 4.16	8.97 ± 2.61	<0.001^∗∗^

Abbreviations: HUA: serum uric acid > 420 *μ*mol/L; Ccr: creatinine clearance; TUG: timed “up and go.” ^∗∗^*p* < 0.001, ^∗^*p* < 0.05.

**Table 4:** Baseline characteristics and laboratory parameters of the study population stratified by T2DM and fracture.

	T2DM (*n* = 1073, 31.3%)			Non-T2DM (*n* = 2357, 68.7%)		
Variables	Fracture (*n* = 78, 7.3%)	Nonfracture (*n* = 995, 92.7%)	*p* value	Fracture (*n* = 123, 5.2%)	Nonfracture (*n* = 2234, 94.8%)	*p* value
Sex, female (%)	56 (71.8)	635 (63.8)	0.157	94 (76.4)	1416 (63.4)	0.003^∗^
Age (years)	70.83 + 6.4	68.95 + 6.53	0.014^∗^	69.55 ± 6.61	68.19 ± 6.18	0.018^∗^
UA (*μ*mol/L)	325 + 83.00	321.08 ± 86.90	0.691	288.65 ± 69.33	312.77 ± 83.66	<0.001^∗∗^
Ccr (mL/min)	70.11 ± 18.94	76.96 ± 21.63	0.007^∗^	74.98 ± 1.8	74.98 ± 19.93	0.983^∗∗^
FBG (mmol/L)	8.03 ± 2.88	7.62 ± 2.39	0.154	5.44 ± 0.56	5.40 ± 0.60	0.449
HbA1c (%)	7.17 ± 1.79	6.95 ± 1.44	0.28	5.43 ± 0.59	5.44 ± 0.58	0.848
25 (OH)D_3_ (ng/mL)	22.68 ± 6.96	22.15 ± 7.68	0.559	22.19 ± 8.51	22.20 ± 7.60	0.98
Ca (mmol/L)	2.42 ± 0.11	2.45 ± 0.09	0.46	2.41 ± 0.09	2.41 ± 0.09	0.298
WHR	0.91 ± 0.06	0.91 ± 0.06	0.374	0.90 ± 0.07	0.88 ± 0.06	0.05
BMD (g/cm^2^)	0.344 ± 0.08	0.36 ± 0.09	0.182	0.32 ± 0.08	0.35 ± 0.08	<0.001^∗∗^
TUG (s)	10.58 ± 5.77	9.76 ± 4.0	0.09	9.86 ± 3.56	8.92 ± 2.54	<0.001^∗∗^

Abbreviations: HUA: serum uric acid > 420 *μ*mol/L; Ccr: creatinine clearance; TUG: timed “up and go.” ^∗∗^*p* < 0.001, ^∗^*p* < 0.05.

**Table 5:** Logistic regression analysis for the risk for fracture in T2DM and non-T2DM subjects, respectively.

	T2DM *N* = 1073 (31.3%)		Non-T2DM *N* = 2357 (68.7%)	
Variables	OR (95% CI)	*p* value	OR (95% CI)	*p* value
Sex, female	1.538 (0.886-2.669)	0.126	1.835 (1.178-2.860)	0.007^∗^
Age	0.987 (0.944-1.031)	0.552	0.969 (0.937-1.003)	0.07
HUA	1.109 (0.507-2.427)	0.795	1.160 (0.547-2.460)	0.698
Ccr (mL/min)	1.010 (0.996-1.025)	0.172	0.993 (0.984-1.001)	0.101
TUG > 10.2 s	1.349 (0.790-2.305)	0.273	1.602 (1.031-2.491)	0.036^∗^
*T*‐score≤−2.5	1.204 (0.744-1.949)	0.449	1.750 (1.196-2.562)	0.004^∗^

Abbreviations: HUA: serum uric acid > 420 *μ*mol/L; Ccr: creatinine clearance; TUG: timed “up and go”; OP: osteoporosis; ^∗^*p* < 0.05.

**Table 6:** The relationship between diabetic parameters and TUG and *T*-score.

Variables	TUG			*T*-score		
	>10.2 s (*n* = 306)	≤10.2 s (*n* = 767)	*p* value	≤-2.5 (*n* = 434)	>-2.5 (*n* = 639)	*p* value
Sex, female (%)	200 (65.4)	391 (64.0)	0.678	295 (68)	396 (62)	0.044^∗^
Age (years)	72.82 ± 6.963	67.6 ± 5.714	<0.001^∗∗^	69.82 ± 6.48	68.59 ± 6.53	0.002^∗^
BMI (kg/m^2^)	26.11 ± 3.54	24.96 ± 3.15	<0.001^∗∗^	25.32 ± 3.47	25.26 ± 3.19	0.762
SBP (mmHg)	145.42 ± 21.34	139.59 ± 19.94	<0.001^∗∗^	141.63 ± 20.44	140.99 ± 20.56	0.614
DBP (mmHg)	79.72 ± 13.79	79.94 ± 11.32	0.783	79.28 ± 12.24	80.29 ± 11.95	0.178
HbA1c (%)	7.05 ± 1.43	6.93 ± 1.48	0.243	6.96 ± 1.41	6.97 ± 1.50	0.92
Ccr (mL/min)	71.17 ± 22.22	78.57 ± 20.87	<0.001^∗∗^	74.45 ± 21.82	77.84 ± 21.20	0.012^∗^
UA (*μ*mol/L)	324.96 ± 89.95	319.94 ± 85.24	0.395	317.31 ± 83.09	324.15 ± 88.88	0.207
TC (mmol/L)	5.05 ± 1.15	5.15 ± 1.07	0.173	5.13 ± 1.08	5.11 ± 1.10	0.785
TG (mmol/L)	1.80 ± 1.01	1.94 ± 1.28	0.085	1.91 ± 1.28	1.88 ± 1.17	0.696
HDL-C (mmol/L)	1.27 ± 0.36	1.32 ± 0.47	0.082	1.32 ± 0.46	1.30 ± 0.43	0.585
LDL-C (mmol/L)	3.15 ± 0.98	3.16 ± 0.91	0.87	3.17 ± 0.93	3.15 ± 0.92	0.782
Ca (mmol/L)	2.42 ± 0.09	2.43 ± 0.09	0.62	2.42 ± 0.09	2.42 ± 0.09	0.961
25 (OH)D_3_ (ng/mL)	21.05 ± 7.73	22.65 ± 7.54	0.002^∗^	22.21 ± 8.04	22.17 ± 7.33	0.93
Hypertension (%)	215 (70.3)	382 (49.8)	<0.001^∗∗^	239 (55.1)	358 (56)	0.757
Therapy of T2DM			0.139			0.681
Oral	65 (21.2)	143 (18.6)		77 (17.7)	131 (20.5)	
Insulin	35 (11.4)	59 (7.7)		41 (9.4)	53 (8.3)	
Oral and insulin	9 (2.9)	24 (3.1)		15 (3.5)	18 (2.8)	
Without medicine	197 (64.4)	541 (70.5)		301 (69.4)	437 (68.4)	

Abbreviations: BMI: body mass index; SBP: systolic pressure; DBP: diastolic pressure; HbA1c: glycosylated hemoglobin; UA: serum uric acid; Ccr: creatinine clearance; TC: total cholesterol; TG: triglycerides; HDL-C: high-density lipoprotein cholesterol; LDL-C: low-density lipoprotein cholesterol; 25 (OH)D_3_: 25-hydroxyvitamin D3; BMD: bone mineral density; TUG: timed “up and go.” ^∗∗^*p* < 0.001, ^∗^*p* < 0.05.

## Data Availability

The cross-sectional data used to support the findings of this study have not been made available because it involves patient privacy.

## References

[1] Cho N. H., Shaw J. E., Karuranga S. (2018). IDF Diabetes Atlas: global estimates of diabetes prevalence for 2017 and projections for 2045. *Diabetes Research And Clinical Practice*.

[2] Yang Y., Guo Y., Qian Z. (. M.). (2018). Ambient fine particulate pollution associated with diabetes mellitus among the elderly aged 50 years and older in China. *Environmental Pollution*.

[3] Wang J., You W., Jing Z., Wang R., Fu Z., Wang Y. (2016). Increased risk of vertebral fracture in patients with diabetes: a meta-analysis of cohort studies. *International Orthopaedics*.

[4] Wang H., Ba Y., Xing Q., Du J. L. (2019). Diabetes mellitus and the risk of fractures at specific sites: a meta-analysis. *BMJ Open*.

[5] Janghorbani M., van Dam R. M., Willett W. C., Hu F. B. (2007). Systematic review of type 1 and type 2 diabetes mellitus and risk of fracture. *American Journal of Epidemiology*.

[6] Palermo A., Tuccinardi D., Defeudis G. (2016). BMI and BMD: the potential interplay between obesity and bone fragility. *International Journal of Environmental Research and Public Health*.

[7] Poiana C., Capatina C. (2017). Fracture risk assessment in patients with diabetes mellitus. *Journal of Clinical Densitometry*.

[8] Vestergaard P. (2007). Discrepancies in bone mineral density and fracture risk in patients with type 1 and type 2 diabetes--a meta-analysis. *Osteoporosis international: a journal established as result of cooperation between the European Foundation for Osteoporosis and the National Osteoporosis Foundation of the USA*.

[9] Podsiadlo D., Richardson S. (1991). The timed “Up & Go”: a test of basic functional mobility for frail elderly persons. *Journal of the American Geriatrics Society*.

[10] Zhu K., Devine A., Lewis J. R., Dhaliwal S. S., Prince R. L. (2011). ‘Timed up and go’ test and bone mineral density measurement for fracture prediction. *Archives of Internal Medicine*.

[11] Jeong S. M., Shin D. W., Han K. (2019). Timed up-and-go test is a useful predictor of fracture incidence. *Bone*.

[12] Cockcroft D. W., Gault H. (1976). Prediction of creatinine clearance from serum creatinine. *Nephron*.

[13] Kanis J. A., McCloskey E. V., Johansson H., Oden A., Melton L. J., Khaltaev N. (2008). A reference standard for the description of osteoporosis. *Bone*.

[14] Zhu K., Lewis J. R., Sim M., Prince R. L. (2019). Low vitamin D status is associated with impaired bone quality and increased risk of fracture-related hospitalization in older Australian women. *Journal of bone and mineral research: the official journal of the American Society for Bone and Mineral Research*.

[15] Chen F., Wang Y., Guo Y. (2019). Specific higher levels of serum uric acid might have a protective effect on bone mineral density within a Chinese population over 60 years old: a cross-sectional study from northeast China. *Clinical Interventions in Aging*.

[16] Zhou Y., Li Y., Zhang D., Wang J., Yang H. (2010). Prevalence and predictors of osteopenia and osteoporosis in postmenopausal Chinese women with type 2 diabetes. *Diabetes Research and Clinical Practice*.

[17] Walsh J. S., Vilaca T. (2017). Obesity, type 2 diabetes and bone in adults. *Calcified Tissue International*.

[18] Piasecki J., Ireland A., Piasecki M. (2019). Comparison of muscle function, bone mineral density and body composition of early starting and later starting older masters athletes. *Frontiers in Physiology*.

[19] Vestergaard P., Rejnmark L., Mosekilde L. (2005). Relative fracture risk in patients with diabetes mellitus, and the impact of insulin and oral antidiabetic medication on relative fracture risk. *Diabetologia*.

[20] Looker A. C., Eberhardt M. S., Saydah S. H. (2016). Diabetes and fracture risk in older U.S. adults. *Bone*.

[21] Melton L. J., Leibson C. L., Achenbach S. J., Therneau T. M., Khosla S. (2008). Fracture risk in type 2 diabetes: update of a population‐based study. *Journal of Bone and Mineral Research*.

[22] Rianon N. J., Smith S. M., Lee M. J. (2018). Glycemic control and bone turnover in older Mexican Americans with type 2 diabetes. *Journal of Osteoporosis*.

[23] Napoli N., Conte C., Pedone C. (2019). Effect of insulin resistance on BMD and fracture risk in older adults. *Journal of Clinical Endocrinology and Metabolism*.

[24] Losada-Grande E., Hawley S., Soldevila B. (2017). Insulin use and excess fracture risk in patients with type 2 diabetes: a propensity-matched cohort analysis. *Scientific Reports*.

[25] Dede A. D., Tournis S., Dontas I., Trovas G. (2014). Type 2 diabetes mellitus and fracture risk. *Metabolism*.

[26] Liu M., Lu Y., Cheng X. (2019). Relationship between abnormal glucose metabolism and osteoporosis in Han Chinese men over the age of 50 years. *Clinical interventions in aging*.

[27] Mousa S. M., Rasheedy D., el-Sorady K. E., Mortagy A. K. (2016). Beyond mobility assessment: timed up and go test and its relationship to osteoporosis and fracture risk. *Journal of Clinical Gerontology and Geriatrics*.

[28] de Abreu D. C. C., Trevisan D. C., Reis J. G., de Carvalho da Costa G., Gomes M. M., Matos M. S. (2009). Body balance evaluation in osteoporotic elderly women. *Archives of Osteoporosis*.

